# Gnathostomiasis Acquired by Visitors to the Okavango Delta, Botswana

**DOI:** 10.3390/tropicalmed5010039

**Published:** 2020-03-06

**Authors:** John Frean

**Affiliations:** 1 Centre for Emerging Zoonotic and Parasitic Diseases, National Institute for Communicable Diseases, Division of the National Health Laboratory Service, Johannesburg 2192, South Africa; johnf@nicd.ac.za; 2 Wits Research Institute for Malaria, University of the Witwatersrand, Johannesburg 2193, South Africa

**Keywords:** *Gnathostoma* species, gnathostomiasis, larva migrans, Okavango, southern Africa, tourists

## Abstract

Gnathostomiasis is a zoonotic nematode parasite disease, most commonly acquired by eating raw or undercooked fish. Although the disease is well known in parts of Asia and Central and South America, relatively few cases have been reported from Africa. Raw fish consumed in the Okavango River delta area of Botswana, and in nearby western Zambia, has previously produced laboratory-proven gnathostomiasis in tourists. The purpose of this communication is to record additional cases of the infection acquired in the Okavango delta, and to alert visitors to the inadvisability of eating raw freshwater fish in the southern African region.

## 1. Introduction

With the global growth in tourism, increasing numbers of travellers visit what were once remote and unusual destinations, many of which are located in tropical, low-income countries. Immersion in local culture can expose travellers to unusual, often unpleasant, and sometimes dangerous pathogens. This is particularly so when they are food- or water-associated, as visitors cannot easily avoid these vehicles of transmission, or may even pursue them in the interests of adventurous eating. Further increasing the risk of infection, local foods may traditionally be consumed raw or incompletely cooked [1]. Importation or local cultivation of exotic food species may greatly extend the geographic range of associated pathogens [2].

**Figure 1:**
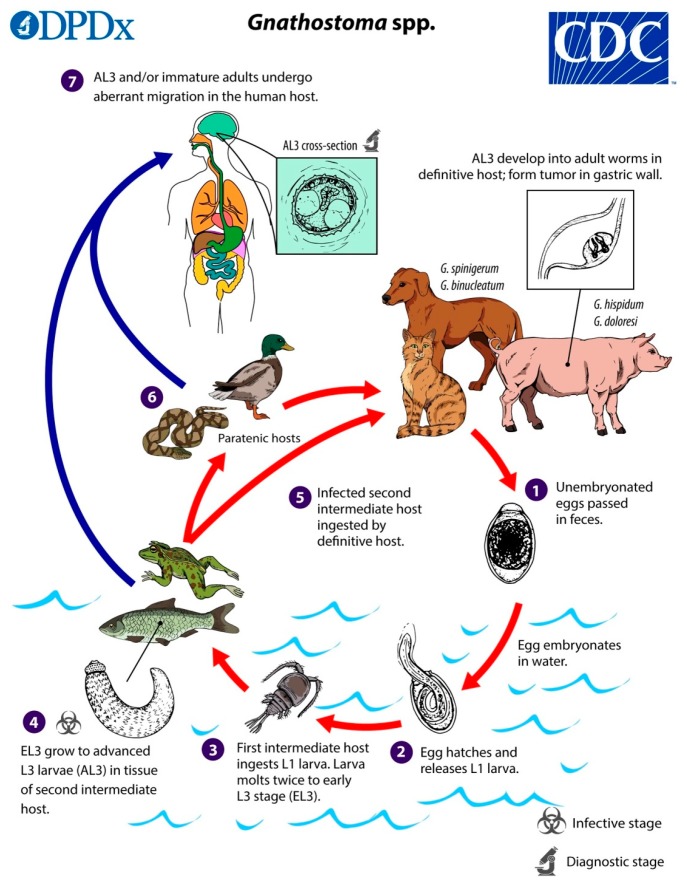
The life cycle of *Gnathostoma* species. (Source: Centers for Disease Control and Prevention, www.dpd.cdc.gov/dpdx/gnathostomiasis/index.html). The adult nematodes occupy the stomach of definitive host animals in the form of a tumour-like mass, and their eggs are passed out in faeces. In water the egg hatches to release a free-swimming ensheathed larva, which after being eaten by the first intermediate host, a small copepod (e.g., *Cyclops* sp.), progresses to the second stage (L2 larva) and early third-stage larva (L3) in its haemocoel. When the infected copepod is ingested by a second intermediate host (usually a fish, of various species, but also crustaceans, eels, frogs, snakes, birds, or mammals), the L3 larva migrates to muscle, and encysts. The late L3 larva traverses the food chain, through predation of paratenic (transport) hosts on one another. In the definitive (final) host, the larva migrates to the stomach via the abdominal cavity and liver, and matures into the adult nematode within about 6 to 12 months. Humans are typically infected when they eat raw or undercooked fish or other intermediate hosts, including eels and crabs; proposed alternative routes of infection are swallowing water containing infected copepods, and direct invasion by L3 larvae through the skin of people handling raw fish or meat [4,5].

*Gnathostoma* species are spirurid nematode parasites with a complex transmission cycle involving terrestrial and aquatic hosts (Figure 1), and humans are infected by consuming the intermediate hosts in the form of raw food, usually fish. While gnathostomiasis is well known in parts of Asia and Latin America [3], it is a relatively newly-described culinary risk in Africa.

**Figure 2:**
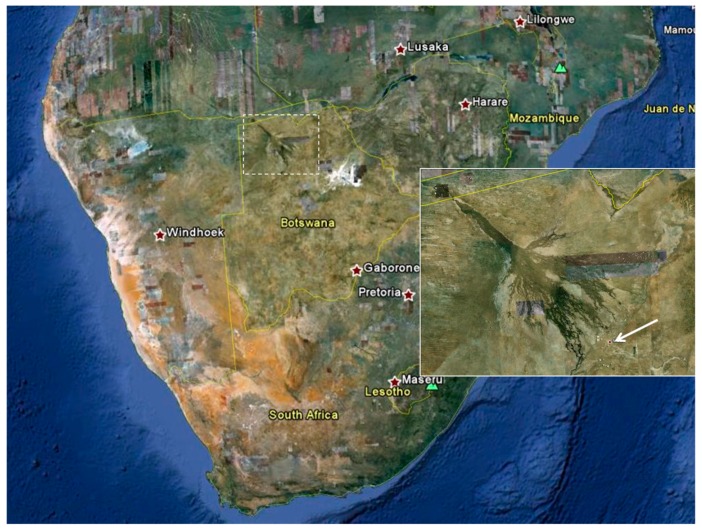
Satellite image of southern Africa. Dashed outline: Okavango delta region in northeast Botswana. Inset: Enlarged view of Okavango delta region showing location of Maun (arrow). (Source: Google Maps, www.google.com/maps/).

This report describes two patients with laboratory-confirmed gnathostomiasis related to raw fish consumption in the Okavango River delta, and a cluster of probable cases with a similar exposure history and suggestive clinical features. The Okavango delta is a unique inland aquatic ecosystem located in northeast Botswana (Figure 2) that attracts many tourists, some of whom are from southern African countries. The purpose of this report is to place these cases of gnathostomiasis on record, and to alert visitors, local residents, and tourism operators to this emerging, potentially serious health risk that is fortunately readily avoidable.

Ethical clearance: All investigation and publication of cases or outbreaks of communicable diseases is carried out by the National Institute for Communicable Diseases under ethical clearance from the Human Research Ethics Committee (Medical) of the University of the Witwatersrand, clearance certificate no. M160667.

## 2. Case Descriptions

### 2.1. Cases 1 and 2

**Figure 3:**
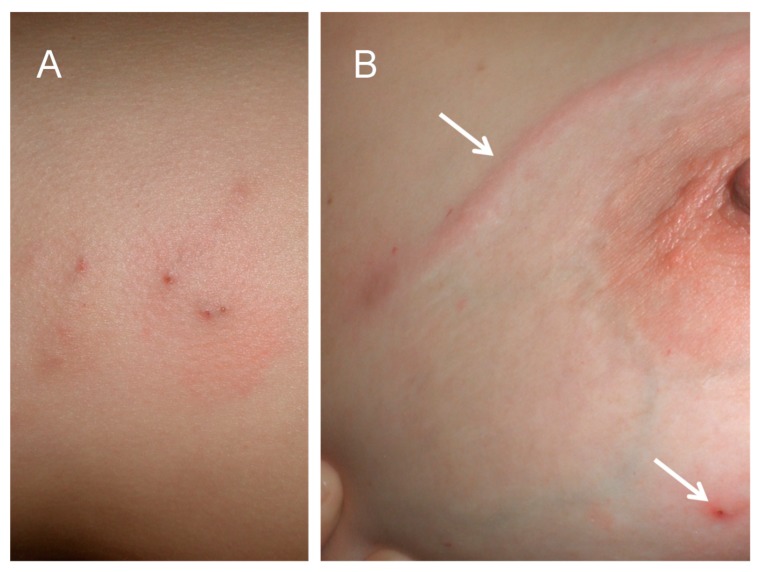
(**A**) Skin nodules caused by localised *Gnathostoma* sp. L3 larvae. (**B**) Linear larva migrans lesion on skin of breast (upper arrow) and skin nodule (lower arrow).

**Figure 4:**
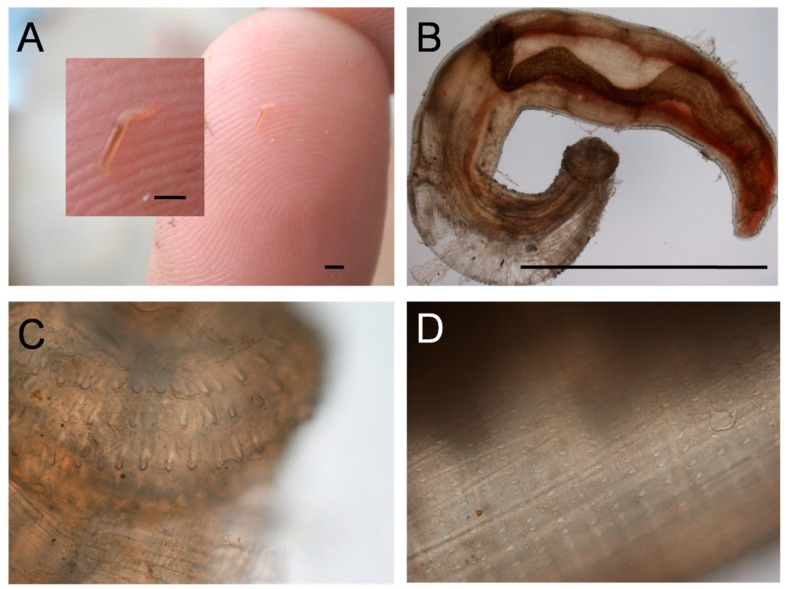
*Gnathostoma* sp. L3 larva. (**A**) As extracted from skin lesion. (**B**) View under ×4 objective. (**C**) Head showing four rows of hooks, ×10 objective. (**D**) Body showing rows of spines, ×10 objective. Bars are approx. 2 mm.

The patients were a middle-aged married expatriate couple living in Maun, Botswana (Figure 2, inset). Both had experienced several episodes of recurrent painful skin nodules (Figure 3A), associated with transient urticarial reactions. The most recent episode had occurred within a few weeks of a trip to the Okavango delta, where (as they usually did on such trips) they had eaten fillets of raw bream (the common name for several indigenous fish species belonging to the family Cichlidae, e.g., *Sargochromis giardi*, *Serrochromis robustus,* and *Coptodon rendalli*) that had been marinated in lemon juice. On this occasion the husband also complained of nonspecific malaise, and the wife developed a painful migratory skin lesion on her left breast (Figure 3B). Some of the nodular skin lesions were superficial, and from several of them, the patients were able to manually express small worms, about 6–10 mm long (Figure 4A). Due to his systemic symptoms, the husband consulted a medical practitioner. Laboratory investigations revealed a haemoglobin level and leucocyte count (16.1 g/dL and 7.5 × 10^9^/L, respectively) within normal ranges, but a high absolute eosinophilia of 1.69 × 10^9^/L (upper limit of normal range is 0.45 × 10^9^/L). Neither the erythrocyte sedimentation rate nor the c-reactive protein level was elevated. One of the worms removed from the skin was preserved in 40% ethanol and sent to the Parasitology Reference Laboratory at the National Institute for Communicable Diseases in Johannesburg, South Africa. Microscopic examination revealed the typical morphological features of the L3 larval stage of a *Gnathostoma* species (Figure 4B,C,D).

### 2.2. Cases 3–5

An outbreak of probable gnathostomiasis occurred among staff and passengers who had been aboard a houseboat that was cruising in the Okavango delta, although laboratory confirmation of these cases is lacking. Four persons on the boat ate freshly-caught raw bream, marinated in lemon juice. Three of the four developed symptoms and signs of gnathostomiasis, including painful migratory subcutaneous lesions. Detailed clinical features are only known for one adult male patient who, five days after eating the raw fish, developed severe diarrhoea and vomiting, followed by headaches and mild fever. Laboratory investigations for malaria and schistosomiasis were negative, and the full blood count and liver function tests were normal. The headaches, fever, and fatigue persisted for more than a week. Eleven days after onset of symptoms, the patient developed severe pain in his right flank and right axilla, spreading posteriorly to the scapula area, suggesting a migratory inflammatory process. On the basis of the geographical, dietary and clinical history, and knowledge of published descriptions of cases acquired in the same area, a clinical diagnosis of gnathostomiasis was made and empiric treatment with albendazole (400 mg daily) was started. Pain and headaches subsided quite quickly, but fatigue persisted until almost the end of the 21-day treatment period. Two other patients with the same symptoms, who respectively received ivermectin and albendazole, also recovered well.

## 3. Discussion

Humans are sometimes accidentally infected with *Gnathostoma* species, which are nematode parasites of fish-eating animals, usually wild and domestic cats and dogs, but the host range of this parasite genus extends to pigs, rodents, raccoons, opossums, otters, weasels and other mustelids, and bears. Among 13 recognised species worldwide, there are at least six that infect humans, the most common of which is *Gnathostoma spinigerum* (reviewed in [1,3]). The species are traditionally differentiated on morphological features, but modern nucleic acid-based methods have also been utilised.

Gnathostomiasis is well known in southeast Asian countries, especially Thailand; the geographic extent includes India, Bangladesh, China, Korea, Japan, and Central and South America (particularly Mexico, Peru, and Equador, countries where ceviche, raw fish marinated in lime juice, is popular) [3,6,7,8]. Africa is a relatively recently recognised risk region for the disease. Three cases from the Rufiji River in southeastern Tanzania have been described (cited in [9]). A man who had lived in South Africa and who presented with eosinophilic oesophagitis, had a positive serological test for *G. spinigerum*, but he had also lived in Southeast Asia, where the disease is common [10]. Previous small outbreaks of gnathostomiasis acquired in the Okavango and nearby western Zambia region have been reported [9,11], but none of these involved South Africans. Captive lions in Namibia and Zimbabwe had eggs of *Gnathostoma* species detected in their faeces by microscopy, additional evidence of endemicity of the parasite in the region [12,13]. The disease is probably more common in humans in southern Africa than is realised, being relatively or completely unknown and therefore not recognised locally; also, laboratory diagnosis is not readily available. The regional epidemiology of human and animal gnathostomiasis, particularly whether the disease affects local inhabitants of the Okavango region, along with surveys of potential intermediate hosts, clearly requires scientific investigation. The southern African cases all acquired the infection by eating raw bream that had been marinated in lemon juice (that is, a version of ceviche), which, according to the patients, is a popular delicacy provided to tourists visiting the delta, suggesting that the risk of acquiring gnathostomiasis continues to exist.

The L3 larvae, measuring up to 12.5 mm in length by 1.2 mm in width, typically migrate through skin and subcutaneous tissues producing painful migratory swellings (the most common presentation). As in the first cases described above, the presentation may be of cutaneous larva migrans (creeping eruption), or localised skin nodules from which the larvae may emerge or be removed. Sometimes larvae invade internal organs including pulmonary, gastrointestinal, or genitourinary systems, or occasionally, the eyes and central nervous system in the most serious forms of the disease. The range of central nervous system disease includes eosinophilic meningitis or meningoencephalitis, radiculomyelitis/encephalitis, and subarachnoid haemorrhage, and may be fatal in up to 25% of cases (reviewed in [7]). Without treatment, the larvae may live for more than ten years. The pathogenesis of gnathostomiasis is related to the mechanical tissue damage produced by the rapid active movement of the migrating larvae, the various secretions and excretions they produce, and the host immune response. Haemorrhagic tracks in the subcutaneous tissue and internal organs typically mark the passage of the larvae [4,7].

Regarding the differential diagnosis, there are several other fish-borne nematode parasites of humans, namely *Anisakis* spp., *Pseudoterranova* spp., *Capillaria philippinensis*, and some other nematode species that very rarely cause disease in humans. However, the clinical presentation of these infections is predominantly gastrointestinal symptoms, unlike gnathostomiasis. Sparganosis, a metacestode disease acquired from raw fish, can also produce soft tissue swellings that are sometimes migratory, but is much slower than gnathostomiasis to develop, requiring years or decades to become clinically evident.

In endemic areas, the diagnosis of gnathostomiasis is usually made by the combination of clinical features and history of consuming undercooked aquatic food items, or sometimes, unusual risk items such as raw snake meat or live fish [1,14]. The diagnosis is supported by the finding of eosinophilia in blood (or cerebrospinal fluid, in the case of central nervous system involvement); however, eosinophilia is not invariable, as in our probable case where the differential leucocyte count was normal. In a British case series of gnathostomiasis in travellers, eight of 16 patients had normal eosinophil counts [11]. Various serological tests have been used, some of which have crossreacted with antibodies against other nematodes. Currently, the preferred laboratory test is immunoblotdetection of a specific 24-kDa antigen produced by *G. spinigerum* L3 larvae, but availability of this investigation is limited to certain tropical disease laboratories in Thailand and Switzerland [7,9].

Effective treatment (albendazole, 400 mg BD for 21 days) for cutaneous infection is available in South Africa. Ivermectin (0.2 mg/kg as a single dose, or 0.2 mg/kg on two consecutive days) is also a suitable anthelminthic drug, but is not routinely available in South Africa. Repeat treatment is sometimes necessary [8]. A sudden rise in absolute eosinophil count may indicate treatment failure. Treating central nervous system invasion is more difficult and corticosteroids might be advisable to reduce the inflammatory response to the parasite [15], but a clinical trial showed no benefit from steroid treatment. In the limited southern African experience of gnathostomiasis to date, there have been no apparent cases of visceral, central nervous system, or ocular involvement.

## 4. Conclusions

Imported gnathostomiasis is an emerging disease resulting from increasing international travel and adventurous eating. Encysted gnathostome larvae in fish are not killed by marinating in lemon or lime juice, salting, or sun-drying. Tourists and recreational fishermen in the Okavango region, as well as other southern African destinations, are advised not to eat raw locally-caught freshwater fish, and tour operators should educate their local guides not to offer this delicacy to tourists.
